# Pain drawings, interpreter support and clinical findings among immigrant patients on sick leave in Swedish primary health care

**DOI:** 10.1017/S1463423619000628

**Published:** 2019-10-04

**Authors:** Monica Löfvander

**Affiliations:** 1 Department of Neurobiology, Caring Sciences and Society, Center for Family Medicine, Karolinska Institutet, Huddinge, Sweden; 2 Center for Clinical Research Västmanland – Uppsala University, Västerås, Sweden; 3 Department of Public Health and Caring Sciences, Family Medicine and Preventive Medicine Section, Uppsala University, Uppsala, Sweden

**Keywords:** chronic pain, depression, immigrants, interpreter, primary care, sick leave

## Abstract

**Aim:**

To evaluate the spread of pain and its correlates among immigrant patients on sick leave.

**Background:**

Backache, outspread pain and sick-leave questions are problematic to handle primary health care, especially in multicultural settings.

**Methods:**

Two hundred and thirty-five patients 20–45 years on paid sick leave (59% women, 93% foreign-born, mostly non-Europeans). Many had little formal education. One-third had professional interpreter support. The patients pointed out on their bodies where they felt pain. This information was transferred on a pain drawing [pain drawing fields (PDFs) 0–18] by a doctor. Major depression and psychosocial stressors were assessed using *Diagnostic and Statistical Manual of Mental Disorders*. Nociceptive locations for pain were established (pain-sites 0–18). Dependent variable was the number of PDFs. Independent variables were social data, sick leave, interpreter, depression, stress levels and number of pain sites. Calculations were done using descriptive methods and multi-variable linear regression in full models, by gender.

**Findings:**

Many patients had depression (51% women versus 32% men). A majority were exposed to psychosocial stressors. Women had more PDFs, in median 5 [inter-quartile ranges (IQR) 4–8] versus men 3 (IQR 2–5), and also more pain sites, in median 3 (IQR 2–5) versus men in median 2 (IQR 1–3). For men, the regression calculations revealed that numbers of PDFs associated only with increasing numbers of pain sites (B 0.871 *P* < 0.001). For women, this association was weaker (B 0.364, *P* < 0.001), with significant values also for age (B 0.103) and sick leave > one year (B 0.767, *P* = 0.010), and a negative predicting value for interpreter support (B −1.198, *P* < 0.043). To conclude, PDFs associated often with somatic findings but varied much among the women. This implies potential problems regarding cause, function and sick leave questions. However, support by professional interpreters may facilitate a shared understanding with immigrant women having long-standing pain.

## Introduction

Chronic backpain, outspread pain and sick-leave issues are problematic to handle in primary care, especially when the objective signs are few or irrelevant for assessing work capacity and when many socio-cultural circumstances are at hand (Wynne-Jones *et al*., 2010; Gerhardt *et al*., 2014; Wainwright *et al*., 2015; Ljungquist *et al*., 2015; Coggon *et al*., 2017). In multicultural health care settings, the various linguistic, social differences and acculturation issues add up in the consultation (De Marchi *et al*., 2018).

Pain and mental ill-health are common reasons for sick leave in younger persons, and especially among women and some immigrant groups (Soares and Jablonska, 2004; Zelkowitz *et al*., 2004). Immigrants and refugees represent a growing portion of patients in primary health care (Allison *et al*., 2002; Brause *et al*., 2012). Both at group and individual levels they will explain ill-health in different ways, thus complicating the diagnostic process of, for example, non-European women with non-malignant widespread pain and long sickness absence (Aznar-Lou *et al*., 2017; Nyen and Tveit, 2018; Wang *et al*., 2019; Benz *et al*., 2019; Eid *et al*., 2019).

To summarise, it can be demanding to diagnose and manage depression and chronic pain in a multicultural context (Nicholl *et al*., 2015). Socio-cultural and linguistic diverse patient populations are common in primary health care around the world. Nevertheless, real-life studies of pain in such primary care settings are few and seldom include if or how professional interpreters influence patients’ presented pain.

This was the background for a study of a new primary care rehabilitation programme based on cross-cultural strategies in a multicultural district in Stockholm, Sweden. The overall aim of the programme was to improve a shared patient–doctor understanding regarding work-hindering pain by using cognitive-behavioural methods focusing on the patients’ fears and attitudes to pain (Löfvander *et al*., 1997).

In this study we aimed to evaluate, by gender, the pain spread shown by the patients in their first encounter with doctors in the rehabilitation programme and the correlates to social and sick-leave data, interpreter support and clinical findings.

## Methods

A real-life study was designed using a doctor’s perspective.

The study was performed in the context of a rehabilitation programme for younger middle-aged employed persons on paid sick leave from 1997 through 2007. The study setting was a primary health care centre in western Stockholm, Sweden, with 15 000 inhabitants (82% foreign born, 120 ethnic minorities, median age 29 years; unemployment rate about 10%). A majority was employed in the service sector and care. The rates of long-term sickness absenteeism and early retirement were high.

### The programme in short

Eligible persons for the programme were the patients 20–45 years of age being on sick leave for at least six weeks for non-malignant disorders. Their doctors at the primary care centre informed the eligible patients of the programme and referred them to it if they consented to participate. Professional interpreters, free-of-charge, using word-to-word translation were available upon request from the patients.

Inclusion was done in consecutive order. Participation in the programme was voluntary.

The essential purpose of the rehabilitation programme was to reduce pain-related fears and to increase self-efficacy. The programme included an introduction consultation with the patient, an interpreter when requested by the patient and two doctors (male and female) taking turns as consultant and observer/secretary. The patient could not choose the sex of the consultant doctor. The four-week subsequent cognitive behavioural treatment consisted of four weekly sessions of patient – consultant doctor discussions about the patients’ ideas about their pain, and 20 daily 1-h general physical training supervised by the physiotherapist. No specific medical or physical treatment was given. The diagnosis of musculoskeletal lesions and depression followed the recommendations given by Swedish health care authorities.

Unemployed patients on long-term social welfare suffering from work-hindering pain were not eligible for this programme.

All three researchers, the two physicians of both sex and the physical therapist, were in principal blinded for all medical data except age and sex. During the first consultation, the physicians worked together, one acting as a consultant, the other as an observer, taking turns in these roles. The observer made field notes in a manual of interview data, clinical findings of somatic and mental ill-health, non-verbal communication of ill-health, and transferred the patients’ shown body parts onto a pain drawing. The observer doctor could also intervene with questions or other examination tests.

The consulting physician had no prior knowledge of the patient and could thus focus on the patient´s own version of his or her illness.

All assessments and diagnoses were made in co-operation between the two doctors. The most pathological alternative was chosen if they differed in their assessments.

### Independent variables

Socio-demographic data included years of schooling, the number of children at home below 18 years of age, country (region) of birth, mother tongue, interpreter support, years since immigration, work and length of prior continuous sick leave. Sick-leave data were also provided by the local health insurance office.

Major depression was diagnosed using the criteria from the *Diagnostic and Statistical Manual of Mental Disorders* (DSM-IIIR) (axis 1 criteria). The first two major criteria must be fulfilled (sadness and loss of interest). These feelings are well known also in a transcultural context and understood also by people who know only little Swedish (Löfvander *et al*., 1997). Further questions about mood changes were asked when we observed sad eyes, and/or slow or agitated psycho-motor activity. Major depression was categorised as existing (yes) or not (no).

Levels of the severity of psychosocial stressors, for example, marital problems, financial strain, trauma, personal illness, children, work, housing and more (1 = mild through 6 = catastrophic) were assessed according to the DSM-IIIR axis 4 criteria.

Pain-related fears (yes; no) were also noted as a separate stressor (see Table 1) when connected to fears of future handicap, death, or another very unpleasant feeling from pain as found from interviews about concepts of pain (Löfvander *et al*., 2007).

We tested for likely nociceptive pain sources from muscular insertion structures at joint-near locations, for example, spine, shoulders, hips (labelled pain sites). A pain site was considered verified when the pain was elicited twice at the same location from isometric muscle contractions against applied resistance (Löfvander *et al*., 1997). The pain sites were noted on a separate sketched human body manual and counted at evaluation. The physiotherapist performed her examination the subsequent day to confirm/add/negate the findings above.

Symptoms and signs of neuropathic pain were also considered. The physical examination included auscultation of heart and lungs, blood pressure and common laboratory tests of blood and urine. Medical records were examined for additional morbidity.

### Dependent variable

All patients, in the upright position wearing underwear, pointed out on their bodies where they felt pain. These body parts were indicated by the observer doctor on the frontal and dorsal views of an empty body manikin and shown to the patient to be approved. The procedure was the same for all patients with respect to the many illiterate patients who did not understand how to use pens, or how to draw. The pain drawings had no axial partitions and were, when evaluated, divided into nine fields each on the frontal and dorsal views. The total number was 18 pain drawing fields (PDFs) consisting of legs and arms, hips, lower back, thoracic back, shoulders, neck-skull on the back side, and in front the face, shoulders, arms, chest, abdomen, lower abdomen, legs. Adjacent pain fields were counted when covered to a minimum of 25% so as to avoid overestimating the dependent variable.

### Statistics

Analyses were performed by gender. Median values with inter-quartile ranges (IQR) were calculated for ordinal and interval data. Numbers and frequencies were calculated for category data. Differences between groups were analysed by chi-square tests (categorical data) or Mann–Whitney U-tests (ordinal and interval data. The dependent variable was the number of PDFs. The independent variables were age 20 through 45 years; number of children at home < 18 years; education as years in school; prior continuous sick leave 1.5–6 months (ref.), 6.1–<12 months (intermediate) or > 12 months (prolonged); European (ref.) and non-European; interpreter: no (ref.) or yes; major depression: no (ref.) or yes; severity of psychosocial stressors 1 (none) through 6 (catastrophic); pain-related fears no (ref.) or yes; number of pain sites 0–18.

We calculated the unstandardised coefficients (B) with 95% confidence intervals (95% CI) for the independent variables as predicting the number of PDFs by using multiple variable linear regression calculation in full models. Model summaries were presented as adjusted R square values by gender.

A two-sided *P*-value < 0.05 was considered as statistically significant. SPSS statistics software version 24.0 was used for the statistic calculations.

### Ethical considerations

The ethical committee of Karolinska Institutet, Stockholm, approved the study (D-no 00-166).

The authors assert that all procedures contributing to this work comply with the ethical standards of the relevant national and institutional guidelines on human experimentation and with the Helsinki Declaration of 1975, as revised in 2008.

## Findings

There were 250 eligible patients. Fifteen of them abstained. Thus 235 (94%) patients (138 women and 97 men) entered the programme (median age 38 years), 82% of them were of non-European birth, mostly from Turkey (women) or Middle East (men) (see Table 1). A minority were born in Southern Europe and a few in Northern Europe (Finland, Poland and England), while six patients were Swedish-born. All were employed, mostly in service and child- or health care. The median residence time was 14 years (range 10–25 years). Yet many patients, especially women, wanted a professional interpreter. In total, one-third of the patients had difficulties to read and write in their mother tongue.

Only a few (9%) had additional medical conditions, for example, mild anaemia, asthma or diabetes, non-significant, between sub-groups. Two men had experienced major traumas. One man had alcohol problems. Most patients used mild analgesics daily, often in small doses. No one reported using morphine-related or psychotropic drugs. No patient had a personality disorder or severe depression, irritable bowel, bladder syndrome, migraine or neuropathic pain.

Common complaints comprised non-radiating backache constraining their ability to work. This pain could include the dorsal pain fields (neck, thoracic and lumbar regions, hips and shoulders) but also a wider spread of pain out to the chest, shoulders, lower abdomen, upper arms or legs. The pain was of superficial character without neuropathic dimensions.

Table 1 shows the descriptive values of the dependent variable and the independent variables for women and men separately. The women had more PDFs than the men but varied much in number: women in median 5 (IQR 4–8) versus men in median 3 (IQR 2–5), *P* < 0.001). They also had significantly more children, shorter education and had more often interpreter support compared to the men. Furthermore, they had more often a major depression (51% versus 32% men). Both women and men were exposed to at least a moderate severity of psychosocial stressors in which pain-related fears were particularly common (81%).

**Table 1.** Characteristics of the study sample by gender

	Women	Men	All
*N*	138 (59)	97 (41)	235
Pain drawing fields^a^ (0–18), md (IQR)	5 (4–8)***	3 (2–5)	5 (3–7)
Age 20–45 years	38 (34–42)	38 (33–42)	38 (34–42)
Children at home, *n* (md (IQR)	3 (1–4)***	2 (0–3)	2 (1–3)
Education (years), md (IQR)	7 (4–11)***	11 (7–12)	9 (5–12)
Sick leave, *n* ( %)			
1.5–6 months	55 (40)	44 (45)	9 (42)
6.1–12 months	23 (17)	15 (16)	38 (16)
>12 months	60 (44)	38 (39)	98 (42)
Non-European, *n* ( %)^b^	113 (82)^b^	79 (81)^c^	192 (82)
Interpreter, *n* ( %)	54 (39)*	26 (27)	80 (34)
Major depression, *n* ( %)^c^	70 (51)**	31 (32)	101 (43)
Severity of psychosocial stressors 1–6, md (IQR)^d^	3 (2–3)	3 (2–4)	3 (2–4)
Pain-related fears, *n* ( %)	110 (84)	81 (80)	191 (81)
Pain sites (0–18), md (IQR)^d^	3 (2–5)***	2 (1–3)	3 (2–4)

md = median; IQR = inter-quartile range.

*P < 0.05, ** *P* < 0.01, *** *P* < 0.001 between women and men.

a Frontal and dorsal pain drawings (nine fields each).

b Fifty-six from Turkey, 28 from the Middle East.

c Criteria from axis I and axis IV of DSM-III-R.

d Reproducible pain at joint-near structures.

Clinical findings show that pain sites were significantly more common among women (md 3; IQR 2–5) than men (md 2; IQR 1–3), *P* = 0.001).

Table 2 shows the full model of the multivariable linear regression models for PDFs, by gender. For men, the regression calculations revealed that increasing numbers of PDFs were significantly associated only with increasing numbers of pain sources (B 0.871; *P* < 0.001). For women, this association was weaker (B 0.364, *P* < 0.001) having significant values also for age (B 0.103) and sick leave > one year (B 0.767, *P* = 0.010), and, notably, also a negative predicting value for interpreter support (B −1.198, *P* < 0.043). Furthermore, women with many children and women of non-European birth had a non-significant trend for higher PDFs. Men with depression had a non-significant trend for higher PDFs.

**Table 2.** Multivariable linear regression in full models as predicting pain drawing fields

	Women			Men		
	B	95 % CI	*P*	B	95 % CI	*P*
Age (20–45 years)	**0.103**	**0.008; 0.199**	**0.035**	−0.051	−0.144; 0.041	0.271
Children at home (*n*)	−0.326	−0.684; 0.032	0.074	−0.107	−0.486; 0.273	0.578
Education (years)	−0.058	−0.195; 0.079	0.405	−0.008	−0.151; 0.136	0.917
Sick leave (1.5–5.9 months; 6–11.9 months; > 12 months)	**0.767**	**0.190; 1.344**	**0.010**	−0.013	−0.637; 0.611	0.966
Non-European (no; yes)	1.275	−0.118; 2.668	0.073	0.157	−1.205; 1.520	0.819
Interpreter (no; yes)	**−1.198**	**−2.357; −0.038**	**0.043**	−0.433	−1.667; 0.801	0.487
Depression (no; yes)	0.556	−0.519; 1.631	0.308	1.182	−112; 2.475	0.073
Psychosocial stressors (1–6)	0.383	−0.210; − 0.976	0.204	0.209	−0.334; 0.752	0.446
Pain-related fears (no; yes)	−0.560	−1.927; 0.807	0.419	−0.597	−2.172; 0.978	0.453
Pain sites (*n*)	**0.364**	**0.189; 0.539**	**<0.001**	**0.871**	**0.594; 1.148**	**<0.001**

Adjusted *R*-square 0.250 full model for women.

Adjusted *R*-square 0.391 full model for men.

Significant values in bold.

Model summaries for PDFs were low for the women, Adj. *R* ^2^ 0.250, and higher for the men, Adj. *R* ^2^ 0.391.

## Discussion

The principal finding was that patients’ pain drawings were mainly associated with the doctors’ somatic findings of pain sites, but this association varied much among the women. The non-European women did not have significantly more PDFs than the other women, and the women, but not the men, with an interpreter support had significantly fewer PDF fields.

### What is new?

To the best of our knowledge, this is an original publication using cross-culturally adapted procedures for evaluating foreign-born patients on sick leave and the influence of interpreter support on their pain reports and their doctors’ diagnostic findings.

### Strengths and limitations

This study had a real-life design and a good external validity but lowered by its cultural and historical frame. Another strength was the homogeneity of this patient sample with persons who shared experiences of migration, the acculturation process, living conditions and chronic pain, and this despite their diverse socio-cultural backgrounds. Most importantly, liberal use was made of professional interpreters, which ensured the trustworthiness of the interview data collected. A limitation is the rather small sample size of patients framed of a rehabilitation programme at a single primary care centre.

Conclusions regarding cause and effect cannot be drawn from this cross-sectional study. A transfer of our results is further limited to younger employed immigrant persons having had a doctor certificate for paid sick leave due to incapacity to work. The results may be possible to transfer to similar multicultural primary care settings where health care professionals meet challenges in cross-cultural medical consultations for pain, depression, distress and sick leave.

### Discussion of methods

Multimodal rehabilitation programmes in primary care tend to exclude women and immigrants because of their assumed poor prognosis (Stenberg *et al*., 2016). In contrast, our study cohort included all eligible patients regardless of sex and socio-cultural background.

Understanding the patients’ complaints is essential in medical consultations (Scheermesser *et al*., 2012) but difficult when the consultations include assessments of functional disability and sickness certification in younger persons with pain and few ‘objective’ signs (Fatahi *et al*., 2005; Roberts *et al*., 2005; Arrelöv *et al*., 2007; Kedzia *et al*., 2015). Reported back and/or shoulder aches or widespread pain is particularly common among immigrants according to Andersson *et al*. (1996) and Gerdle *et al*. (2008). They are often also exposed to excess working place strains and hazards (Engkvist *et al*., 2000; Kamaleri *et al*., 2009; Overland *et al*., 2012). Our study tried to address these issues by designing a real-life study focusing on diagnostic findings and language support in younger immigrant patients with non-malignant pain being certified sick from work for lengthy periods.

Various linguistic, cultural and educational inequalities had to be overcome in this study. To secure a valid and reliable data collection we had Swedish doctors of both sex, had sign-language as a standard to indicate pain spread on the body, provided free availability to professional interpreters regardless of fluency in Swedish, but also encouraged the use of basic Swedish words. The DSM criteria for evaluating depressive symptoms have proved both valid and reliable across cultures (DSM-IIIR). The two major symptoms were nearly always uncomplicated to ask for in an everyday consultation manner whether using basic Swedish or via an interpreter.

### Discussion of results

Here, presented pain and somatic findings varied more among the women than they did among the men. Distress and depression did not sufficiently account for this.

Most patients presented pain in the back and shoulder region. This backside pain was mainly understood from a doctor’s perspective as being of somatic origin also in the non-European women. Our patients’ focus on pain in the back contrast to the principally frontal pain in ‘half-body pain’ (Bäärnhielm and Ekblad, 2000; Löfvander *et al*., 2007), or the widespread ‘overall’ pain described by women and suggested to be related to distress, evaluation of stimuli, pain coping and/or hormonal effects (Bergman, 2005; Kindler *et al*., 2011; Tsay *et al*., 2015).

An encouraging assumption from the result here is that interpreter support by professionals seems to have been of a substantial help for patients to verbalise their pain complaints, thus enabling a patient–doctor understanding of special importance for patients with little education, widespread pain and more severe mental ill-health (Fatahi *et al*., 2005; Fryer *et al*., 2012; Falla *et al*., 2016).

Persons with fibromyalgia tended to have a higher psychosocial burden compared with those having widespread pain only (Gerhardt *et al*., 2017). Both conditions were common among immigrants (Bergman, 2005). Only one patient here had fibromyalgia while it was more common that patients had an un-specific widespread pain and depression resembling the criteria for somatoform (idiopathic) pain syndrome (DSM-III-R).

A perceived widespread pain might shrink by time (Papageorgiou *et al*., 2002). Contrasting to this are the many studies describing extended sick-leave periods with disability benefits to associate with more outspread pain possibly by inactivity, but also by many ‘learning’ occasions when consulting various medical professionals (Kindler *et al*., 2010; Pienimäki *et al*., 2011; Gerhardt *et al*., 2014; Falla *et al*., 2016). With this in mind, we noted that no unemployed immigrant patient presented outspread pain despite having long-term disabling pain but without a doctor sickness certificate (personal communication from the parallel rehabilitation programme).

Here, increasing PDFs did not associate with major depression or more severe stress among women, with a question mark for the men. This corresponds with findings among Nordic citizens, of pain as not linked to depressive symptoms alone (Kamaleri *et al*., 2009; Kindler *et al*., 2010). In contrast, other authors found widespread pain to be more common in distressed, traumatised or mentally ill persons (McBeth *et al*., 2001). Also learning and iatrogenic factors, secondary gains, illness behaviours and family roles may contribute for the pain to extend into multiple fields (Nordeman *et al*., 2012; Overland *et al*., 2012; Scheermesser *et al*., 2012). Other contributing factors to pain spread might be a disturbed central pain modulation, hypersensitivity and awareness of bodily sensations (Harkness *et al*., 2005; Kindler *et al*., 2010; Schliessbach *et al*., 2013; Gruszka *et al*., 2018). Interestingly, a low alcohol intake seems to relate to a wider spread of pain (Arvidsson *et al*., 2008). To compare, most of our patients had manual jobs and pain-related fears, but no joint disorders, and were not hypersensitive to touch but they reported a low or very low consumption of alcohol. Furthermore, the additional somatic complaints to pain were few here, in contrast to the features of widespread pain in medically unexplained syndromes (Burton, 2003; Nordeman *et al*., 2012).

### Implications for clinical care

Professional interpreters may facilitate to create a patient-doctor bridge which could be of special importance for women with little formal education. Such a strategy may reduce the gap between immigrant patients´ described pain and diagnostic findings, thus making it easier to establish diagnoses, assess functional disability and find treatment strategies for return to work. Our results can be valid also today since mental and musculo-skeletal disorders are still major reasons for disability among younger persons, and medical professionals still need helping strategies in cross-cultural medical encounters.

### Future studies

Further studies are warranted regarding the role of professional interpreters in medical consultations regarding patients with pain and work disability. Such study designs require funding bodies to recognise the importance of including linguistically diverse people in the participant samples.

## Conclusion

The patients indicated a spread of pain that was mainly associated with diagnostic findings of pain sites with larger variations among the women. Non-European women did not differ from this pattern. Women supported by professional interpreters indicated fewer pain fields. On the whole, the findings imply potential counselling problems in multicultural health care regarding cause, treatment and in the sick-leave issuing process. Further research and development studies are warranted in multicultural primary care settings for younger people with chronic pain and disability.
